# Pathophysiological role of host microbiota in the development of obesity

**DOI:** 10.1186/s12937-016-0166-9

**Published:** 2016-04-23

**Authors:** Nazarii Kobyliak, Oleksandr Virchenko, Tetyana Falalyeyeva

**Affiliations:** 1 Bogomolets National Medical University, T. Shevchenko Boulevard, 13, Kyiv, 01601 Ukraine; 2 Taras Shevchenko National University of Kyiv, Volodymyrska Str., 64/13, Kyiv, 01601 Ukraine

**Keywords:** Obesity, Gut microbiota, Intestinal permeability, Innate immunity, Metabolic inflammation, Endocannabinoid system, Bile acid metabolism, FXR, Short-chain fatty acids, TLRs, FIAF

## Abstract

Overweight and obesity increase the risk for a number of diseases, namely, cardiovascular diseases, type 2 diabetes, dyslipidemia, premature death, non-alcoholic fatty liver disease as well as different types of cancer. Approximately 1.7 billion people in the world suffer from being overweight, most notably in developed countries. Current research efforts have focused on host and environmental factors that may affect energy balance. It was hypothesized that a microbiota profile specific to an obese host with increased energy-yielding behavior may exist. Consequently, the gut microbiota is becoming of significant research interest in relation to obesity in an attempt to better understand the aetiology of obesity and to develop new methods of its prevention and treatment. Alteration of microbiota composition may stimulate development of obesity and other metabolic diseases via several mechanisms: increasing gut permeability with subsequent metabolic inflammation; increasing energy harvest from the diet; impairing short-chain fatty acids synthesis; and altering bile acids metabolism and FXR/TGR5 signaling. Prebiotics and probiotics have physiologic functions that contribute to the health of gut microbiota, maintenance of a healthy body weight and control of factors associated with obesity through their effects on mechanisms that control food intake, body weight, gut microbiota and inflammatory processes.

## Introduction

Today obesity has become pandemic; about 1.7 billion people on the planet are overweight. World Health Organization has declared obesity a global epidemic and has initiated efforts to control it. The obesity epidemic is a result of changes in energy intake and/or energy expenditure that have led to energy imbalance in a large portion of the population [1]. Because environmental changes lead to such changes through altered behaviors, researchers have been focused on eating patterns, physical activity, and sedentary behaviors. However, these behaviors are likely to vary between population groups, including based on gender and age differences. However, it is known that different dietary regimens can affect body weight. For example, increased fruit and vegetable intake results in greater reduction of weight than limited intake of high-fat low-nutrient dense foods with controlled physical activity [2].

Overweight and obesity increase the risk for cardiovascular diseases, type 2 diabetes (T2D), dyslipidemia, premature death, hepatobiliary disease (non-alcoholic fatty liver disease, gallbladder dyskinesia, cholelithiasis) as well as lung, breast, uterine and ovarian cancer. The permanently growing cohort of patients with obesity-related diseases requires an urgent change of paradigm from interventional measures to predictive, preventive and personalized medicine.

The human body is not only a complex group of organs and systems, but also contains more than 500 different species of microorganisms that accompany human from birth to death [3]. Human biological entity is a stable symbiosis of two equal autonomous systems: macroorganism (host) and symbiotic microorganisms that are evolutionarily adapted to life in relatively open human organs on the basis of mutually beneficial relations [4, 5]. During phylogenesis, symbiosis of host and microflora was steadily improving, resulting in transformation of microbiota into a kind of vital regulatory body [6], consisting of a large number of microbial cells, the number of which is 1–3 times higher than the number of own human cells [7–9]. This “organ” has a wide range of functions that are vital for whole body. Microorganisms that are routinely found in healthy people considered to the normal microbiota, which is defined as a set of populations of microbes in individual organs and systems in certain qualitative and quantitative ratios that support the host organism’s biochemical, metabolic and immunological balance necessary for health maintenance. [10]

Human microbiota includes hundreds of different species with a total number of the cells over 10^11^–10^13^. Moreover, microorganism species composition depends on the organ inhabited [11]. The largest number of microorganisms is in the habitats of the digestive tract. Each part of the digestive system is characterized by different composition of microbial flora (Table 1) [12, 13]. However, the most simple method to count the bacteria number is the investigation of fecal samples and this does not fully reflect the microbiota content throughout the digestive system. So, the true composition of microflora and its functions may be misleading. Additionally, the data from different studies vary because of a great inter-individual difference in microflora [13].

**Table 1:** The content and composition of microflora in different parts of the human digestive tract in health

Habitats of the digestive tract	The number of microorganism cells per 1 g of content		Dominant microflora
	Lumen microflora	Surface microflora	
Mouth	10^8^–10^9^	10^11^–10^12^	*Streptococcus* (60–90 %), *Lactobacillus, Bifidobacterium, Propionibacterium, Bacteroides, Actinomyces*
Stomach	10^2^–10^3^	10^5^–10^6^	Acid resistant *Lactobacillus, Streptococcus, Staphylococcus*
Proximal small intestine	10^3^–10^5^	10^10^–10^11^	*Streptococcus, Lactobacillus, Enterococcus, Bifidobacterium, Escherichia,*
Distal small intestine	10^8^–10^10^	10^10^–10^12^	*Lactobacillus, Escherichia, Enterococcus, Bacteroides, Bifidobacterium*
Colon	10^11^–10^12^	10^10^–10^12^	*Bifidobacterium*, *Lactobacillus*, *Propionibacterium*, *Bacteroides – 90*–*95 %, Escherichia, Enterococcus – 5*–*10 %*

The most studied part of digestive tract regarding microflora is colon which characterized by the largest variety of microorganisms [7, 14]. The dominant species of obligate microflora are asporogenous gram-positive and gram-negative saccharolytic anaerobes: *Bifidobacterium*, *Lactobacillus*, *Propionibacterium*, *Bacteroides. Bifidobacteria* and *Bacteroides* comprise 85–98 % of intestinal microflora (Table 1) [15].

## Review

### Altered composition of gut microbiota in obesity

Recent evidence suggests that gut microbiota is involved in the control of body weight, energy homeostasis and inflammation, and thus plays a role in the pathophysiology of obesity. Prebiotics and probiotics are of interest because they have been shown to alter the composition of gut microbiota and to affect food intake, appetite, body weight and composition as well as metabolic functions through gastrointestinal pathways and modulation of the gut bacterial community [16].

At present, the question of the probiotics’ influence on lipid metabolism and obesity is actively debated in the scientific literature [17–19]. Backhed et al. were the pioneers in the study of the role of colon microflora in regulation of metabolism [20]. Their findings were the catalyst for progress in this field. Further studies have shown that the composition of intestinal microbiota is altered in overweight people. Thus, intestine microbiocenosis can be considered the environmental factor that modulates the development of obesity. It was demonstrated that prolonged exposure to a high fat diet (HFD) significantly changed the composition of the colon microflora in mice, leading to a reduction in the levels of *Bifidobacterium* and *Lactobacillus* that are known to produce many positive physiological effects, e.g. improving the barrier function of the intestinal mucosa as well as to an increase in the levels of *Firmicutes* and *Proteobacteria* that include pathogenic species [19, 20]. Different studies have shown the decrease of abundance of Bacteroides (phylum *Bacteroidetes*) and increase of *Bacillaceae*, *Clostridiaceae* and other representatives of phylum *Firmicutes* [21, 22]. Others speculate that not the ratio of Firmicutes and Bacteroidetes is important in obesity but emphasize on altered proportions of *Actinobacteria* in obese individuals [23]. It is also reported that gut of obese people is greatly inhabited with H_2_-oxidizing methanogenic *Archaea* [24]. It is supposed that these microorganisms oxidize H_2_ produced by H_2_-producing bacteria from *Prevotellaceae* family (phylum *Bacteroidetes*). Rapid H_2_-utilization accelerates fermentation of polysaccharides by *Prevotellaceae* and consequently results in the more considerable energy uptake by obese individuals [24].

The importance of microbiota modification in the conditions of obesity is confirmed by numerous studies of the probiotic interventions (Table 2). The analysis of more than 20 articles from 2013 to July 2014 by Cani et al. showed that at least 15 different strains of *Lactobacillus* and two strains of *Bifidobacterium* do not equally influence on body weight, fat mass, glucose metabolism, inflammatory markers, plasma and hepatic lipids and plasma cholesterol levels [25]. Furthermore, no single strain had all of these effects on different models of obesity in rats. In our research the combination of two *Bifidobacterium* and one *Lactobacillus* lyophilized strains did not influence body mass index and Lee index. At the same time, they strongly reduced fat mass and serum lipids in rats and improved hormonal activity of adipose tissue, thus demonstrating the more pronounced combined effect on obesity as compared to the effects of single strains described in the aforementioned article [25].

**Table 2:** The alteration of microbiota in gut in the conditions of obesity

Phylum	Class	Order (Genera)	The trends of changes	Reference
Bacteroidetes	Bacteroidetes	Bacteroidales (Bacteroides)	↓	[20, 110, 111]
		Bacteroidales (Prevotella)	↑	[24, 110]
Firmicutes	Bacilli	Bacillales (Bacillus)	↑	[19]
		Lactobacillales	↓	[20]
	Clostridia	Clostridiales (Clostridium)	↑	[23, 112]
Actinobacteria	Actinobacteria	Actinomycetales	↑	[23]
	Actinobacteria	Bifidobacteriales (Bifidobacterium)	↓	[12]
Euryarchaeota (domain Archaea)	Methanobacteria		↑	[24]

### Gut microbiota and obesity: pathways and mechanism of interactions

The mechanisms of obesity development and microbiota impact on it are under the close attention of scientists. The most frequent cause leading to the obesity development is a dysbalance between energy intake and energy expenditure. In this complex process, genetic susceptibility, environmental and lifestyle factors are involved. Recent advances in next generation sequencing technology and mechanistic testing in gnotobiotic mice have identified the gut microbiota as an environmental factor which influences whole-body metabolism [26]. Gut microbiota affect energy balance, inflammation state and gut barrier function, as well as integrate peripheral and central food intake regulatory signals leading to an increase in body weight. Underlying mechanisms of the gut microbiota contribution to host metabolism were revealed from studies on germ-free mice which were protected against developing diet-induced obesity.

### Fasting-induced adipose factor (FIAF)

**Fig. 1:**
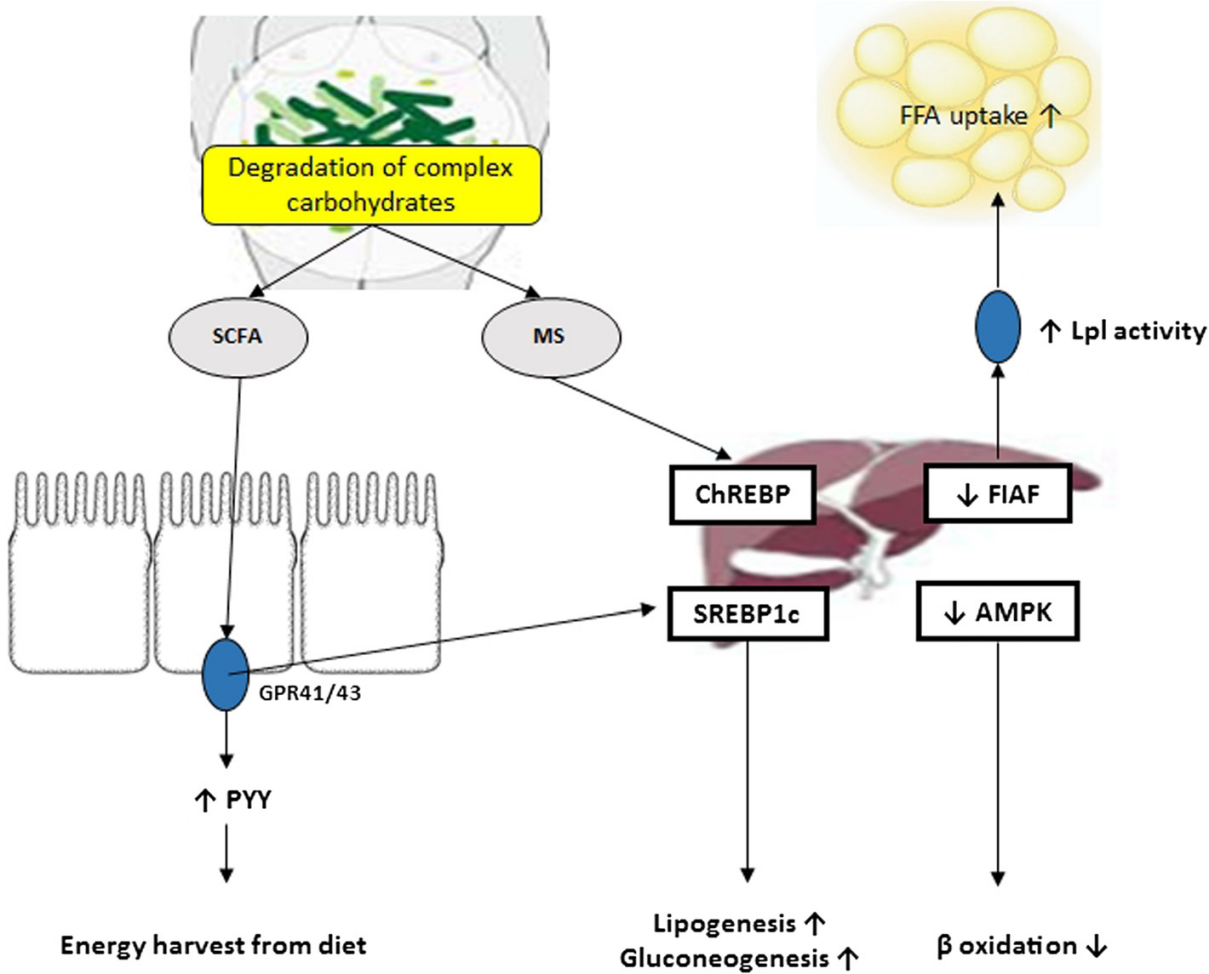
Mechanism linking altered gut microbiota to obesity

One of the key mechanisms by which germ-free animals are protected from diet-induced obesity is elevated levels of fasting-induced adipose factor (FIAF), also known as angiopoietin-like protein 4. FIAF is a circulating lipoprotein lipase (Lpl) inhibitor produced by the intestine, liver and adipose tissue [27]. Conventionalization of germ-free mice suppresses expression of *Fiaf* in the gut epithelial cells [20]. This leads to a higher adipocyte Lpl activity and results in increased cellular uptake of fatty acids, adipocyte triglyceride accumulation and greater fat storage (Fig. 1). Germ-free *Fiaf–/–* mice are obese similarly to their conventionally reared counterparts. After conventionalization, germ-free *Fiaf–/–* mice had a 57 % higher total body fat than their wild-type littermates [20]. Consistently, germ-free *Fiaf–/–* mice fed a high-fat high-carbohydrate diet were not protected from diet-induced obesity, suggesting that FIAF is a mediator of microbial regulation of energy storage [28].

In contrast, mice fed a high-fat diet complemented with *Lactobacillus paracasei* exhibited significantly reduced body fat, which was paralleled by increased circulating levels of FIAF [29]. Fleissner et al. showed that germ-free mice on a high-fat diet showed increased intestinal mRNA expression of *Fiaf* with no major changes in circulating FIAF, as compared to conventionalized mice, suggesting that FIAF mechanism is not universally associated with gut microbiota-related fat mass development [30].

### AMP-activated protein kinase (AMPK)

Furthermore, Backhed and colleagues have also demonstrated that germ-free mice exhibit increased levels of phosphorylated AMPK in muscle and liver. AMPK is a key enzyme that controls cellular energy status, which in turn activates key enzymes of mitochondrial fatty acid oxidation, including acetyl-CoA carboxylase (ACC) and carnitine-palmitoyltransferase I (CTP1) (Fig. 1). This enzyme activation is indicative of increased energy expenditure. The exact pathway through which the microbiota signals to liver and skeletal muscle AMPK is unclear, but appears to be independent from FIAF [28].

### Intestinal microbiota, short-chain fatty acids and energy harvest from the diet

An important role of intestinal microbiota is synthesis of various biomolecules. For instance, microflora produces wide range of vitamins (C, B, folate and niacin) and essential amino acids and facilitates their absorption [31]. Flora also promotes better absorption of calcium and vitamin D [32]. Anaerobic bacteria synthesize biologically active substances: β-alanine, 5-aminovaleriс and γ-aminobutyric acid [33, 34]. Normal flora of the human body participates in the metabolism of proteins, carbohydrates, lipids and nucleic acids; breaks down cellulose; provides epithelium with substrates of gluconeogenesis and lipogenesis; and stimulates intestinal motility [35].

The gut microbiota that digests complex dietary carbohydrates produces many monosaccharides and short-chain fatty acids (SCFAs) such as acetate, propionate, and butyrate [28] which are an important energy source and nutrition of the intestinal epithelium. Additionally, gut microbes enhance the intestinal barrier and help eliminate potential pathogens [36]. Conventionalization of germ-free mice doubles the density of small intestinal villi capillaries [37] and enhances an uptake of these components from the gut into the portal blood and eventually participates in hepatic *de novo* lipogenesis promoting fat accumulation in the liver and adipose tissue [28]. This reaction is controlled by carbohydrate responsive element binding protein (ChREBP) and sterol responsive element binding protein (SREBP-1) [38]. Furthermore, monosaccharides that are produced by microbial fermentation and absorbed and transferred to the liver via portal vein, activate ChREBP which increases the transcription of several proteins involved in hepatic de novo lipogenesis [39]. This contributes to hepatic steatosis.

SCFAs act in the gut as signaling molecules and are specific ligands for at least two G protein-coupled receptors, GPR41 and GPR43, mainly expressed in intestinal epithelial cells [39, 40]. Samuel et al. have demonstrated that conventionally raised *Gpr41*–/– mice and germ-free *Gpr41–/–* mice colonized with only *Bacteroides thetaiotaomicron* and *Methanobrevibacter smithii* are significantly leaner than wild-type littermates, while there are no differences between wild-type or *Gpr41–/–* germ-free mice [41]. Gpr41, which is produced by enteroendocrine cells, may be a regulator of host energy balance through effects that are dependent on gut microbiota (Fig. 1). Activation of GPR41 increases production of peptide YY (PYY), an enteroendocrine cell hormone that normally inhibits gut motility, increases intestinal transit rate and reduces extraction of energy from the diet, thus affecting peripheral glucose utilization [41]. Recent study has shown that *Gpr43–/–* mice are resistant to diet-induced obesity and insulin resistance, at least partly due to Gpr43-regulated energy expenditure [42].

### Innate immunity and metabolic inflammation

Intestinal epithelium is the largest surface of cross-talks with gut microbes. Innate immune system of the intestine is one of the most important factors involved in the interaction between microflora and the host. This symbiosis can on the one hand lead to the destruction of pathogenic microorganisms, while at the same time promoting tolerance to commensal, thus creating ecological niches for useful and consistently associated with the gut microorganisms [43, 44].

The host symbiotic bacteria realize the effect on the immune system through the interaction between their pathogen-associated microbial patterns (PAMP) (including lipopolysaccharide (LPS), lipoteichoic acids (LTK) of cell walls of bacteria, flagellin and double- or single-stranded RNA and DNA) and specific toll-like receptors (TLRs) of epithelial and dendritic cells (DCs) of the digestive tract [43, 45, 46]. TLRs are family of integral membrane pattern-recognition receptors that have a crucial role in the innate immune system and are important for maintaining this balance [47]. Bacterial cells are recognized by the host in three ways: 1) interaction with TLR on DC projection on the surface of the mucosa; 2) interaction with TLR on DC in subepithelial layer after the translocation of bacteria through M cells in lymph plaques without degradation; and 3) binding to enterocyte receptor and subsequent PAMP presentation to DC [45]. Binding of PAMP leads to connection of adaptor protein myeloid differentiation primary response gene (MyD88) to TLR. Another domain of this protein interacts with the interleukin 1 receptor associated kinases (IRAK): IRAK1 and IRAK4. IRAK4 phosphorylates IRAK1, which allows joining of another adaptor protein TNF receptor associated factor 6 (TRAF6). TRAF6 is associated with mitogen-activated protein kinase (MAP2K), transforming growth factor (TGF) -β-activated kinase (TAK) 1, TAK-binding protein 1 (TAB1) or NF-κB-inducing kinase (NIK). As a result, the phosphorylation and activation of IκB kinase (IKK) that phosphorylates inhibitor of NF-κB IκB takes place. This provides the release of NF-κB, which migrates to the nucleus and triggers the transcription of various cytokines, chemokines, adhesion molecules, and acute phase proteins, for instance IL-1β, IL-6, and IL-8. In most cells, the activation of NF-κB inhibits apoptosis [45].

It should be noted that generally commensal bacteria do not cause inflammation through hyperactivation of the immune system. This is due, firstly, to the lack of microflora-produced PAMP, and secondly, to normal expression of TLR3 and TLR5 and poor expression of TLR2 and TLR4 by enterocytes in the normal state. Lack of TLR2 and TLR4 is a possible explanation for the insensitivity of intestinal cells to LPS of commensal bacteria, but the presence of TLR3 and TLR5 causes sensitivity of epithelium to infection through flagellated bacteria and components of enteropathogenic bacteria [48, 49]. In support of this, Furrie et al. showed that TLR2 and TLR4 expression was observed only in the crypts. As epithelial cells mature and migrate to the villi surface, expression of these receptors decreases [50]. In addition, intestinal epithelial cells express a large quantity of TLR-inhibiting peptide (TOLLIP), which inhibits TLR2- and TLR4-mediated pathways and thus protects host organism from a chronic inflammatory response to commensal bacteria [51]. Another mechanism for maintaining tolerance to symbiotic bacteria is the microbial ability to reduce ubiquitination of IκB, which reduces its destruction and prevents NF-κB translocation to the nucleus and consequent activation of pro-inflammatory genes [52].

Cani et al. demonstrated that bacterial LPS, which is continuously produced in the gut through a lysis of gram-negative bacteria, is a microbiota-related factor that can trigger an inflammatory process by binding to the CD14/TLR-4 complex at the surface of innate immune cells [53]. Author mentioned that after 4 weeks of high-fat feeding, mice exhibited an obese phenotype accompanied by a change in gut microbiota composition (reduction of *Bifidobacteria* and *Eubacteria spp*.) and a 2–3 fold increase in circulating LPS levels, which they called “metabolic endotoxemia” since LPS plasma concentrations were much lower than those observed during septic shock [54]. In fact, in this study, continuous subcutaneous low-rate infusion of LPS led to excessive weight gain and insulin resistance in mice. Moreover, mice deficient in LPS receptor (*Cd14-/-*) tend to be resistant to this chronic inflammatory state and are hypersensitive to insulin even when they are fed a normal diet, suggesting that CD14 may modulate insulin sensitivity under physiological conditions [55]. Deletion of TLR-4 prevents the HFD–induced insulin resistance [56]. Molecular links leading to TLR4-induced insulin resistance are not fully elucidated, but some studies mention that TLR4 signaling interferes with insulin signaling. Furthermore, a stimulation of TLR4 by fatty acids can lead to the recruitment of pro-inflammatory macrophages to adipose tissue [57, 58] and cross-talk between macrophages and adipocytes in adipose tissue, which involves activation of NF-κB and JNK by TLR signaling and mediates insulin resistance by phosphorylation of IRS-1 [59, 60].

In recent study, Csak et al., demonstrated that knockout of *Tlr4* protected mice from fibrosis development and lead to a significant attenuation of steatohepatitis and a decrease in serum alanine transaminase levels and oxidative stress [61].

Another member of pattern-recognition receptors family, TLR5, may be associated with an altered gut microbiota metabolic changes development in the host. *Tlr5*-deficient mice exhibit hyperphagia and develop hallmark features of metabolic syndrome, including hyperlipidemia, hypertension, insulin resistance, and increased adiposity. All of these phenotypes are associated with altered gut microbiota composition. Furthermore transplantation of microbiome from *Tlr5*-deficient mice to WT germ-free mice conferred many features of metabolic syndrome to the recipients [62].

TLR-2 recognizes components of gram-positive bacterial cell wall, such as peptidoglycan and lipoteichoic acid. In methionine-choline deficient (MCD) diet-induced model of NASH the role of TLR-2 has been examined. *Tlr-2* deficient mice demonstrate significantly higher steatosis, inflammation and necrosis histological score as compared with WT littermates. Furthermore, they exhibite an increase in liver injury associated with approximately 3-fold elevation of TNF-α mRNA expression. Possibly, the TLR-2 deficiency exacerbates NASH by altering signaling via the TLR-4 pathway due to their polymorphism [63, 64].

TLR-9 is a pattern recognition receptor that recognizes bacteria-derived cytosine phosphate guanine (CpG)-containing DNA and can be involved in the pathogenesis of NAFLD. TLR9- and MyD88- (adaptor molecule for TLR9) deficient mice have significantly lower insulin resistance and show less steatohepatitis and liver fibrosis histological pattern than WT mice. TLR9 signaling induces production of IL-1β by Kupffer cells and therefore increases lipid accumulation in hepatocytes, which leads to the NF-kB inactivation, resulting in cell death [65].

It has also been demonstrated that modulation of gut microbiota (e.g. by an antibiotic treatment and probiotics or dietary intervention with oligofructoses) reduces metabolic endotoxemia and the cecal content of LPS, improves glucose intolerance, insulin sensitivity and decreased body weight gain, and prevents development of obesity and NAFLD both in animal models of obesity and in human studies [66–70].

A recent study [71] examined the possibility that MyD88, a central adaptor molecule for the majority of TLRs, acts as a sensor in the interaction between gut microbes and the host in obesity. Specific tamoxifen-induced *MyD88* deletion in intestinal epithelial cells protects against diet-induced obesity, is associated with increased energy expenditure, improves glucose homeostasis, and reduces hepatic steatosis and whole-body fat mass by 30 %. *MyD88* deletion protects mice against HFD-induced metabolic endotoxemia, thereby supporting the hypothesis that the deletion improves metabolic inflammation. Gut microbiota transplantation from *MyD88-KO* HFD mice into germ-free recipient mice fed a HFD or into mice with intestinal MyD88 deletion after the onset of obesity reduces body weight gain, fat mass development and adipose tissue inflammation, indicating that targeting intestinal epithelial MyD88 constitutes a putative therapeutic approach for obesity and associated disorders.

Kleinridders et al. demonstrated that mice with *MyD88* deletion in the central nervous system are protected from HFD-induced weight gain, leptin resistance and from the induction of leptin resistance by acute central application of palmitate [72]. Conversely, according to Everard study, a key mechanism leading to protection against diet-induced obesity is change in food intake, which is independent from energy intake and energy absorption. These data suggest that the impact of *MyD88* deletion on energy intake is tissue-dependent: in the central nervous system MyD88 controls leptin sensitivity and appetite via fatty acid signaling, whereas in the intestine MyD88 controls energy metabolism via cross-talk with gut microbes [71].

Additionally, the activation of MyD88-independent signaling pathway can lead to early induction of IFN-β, as well as to activation of IFN-induced genes such as iNOS [45].

**Fig. 2:**
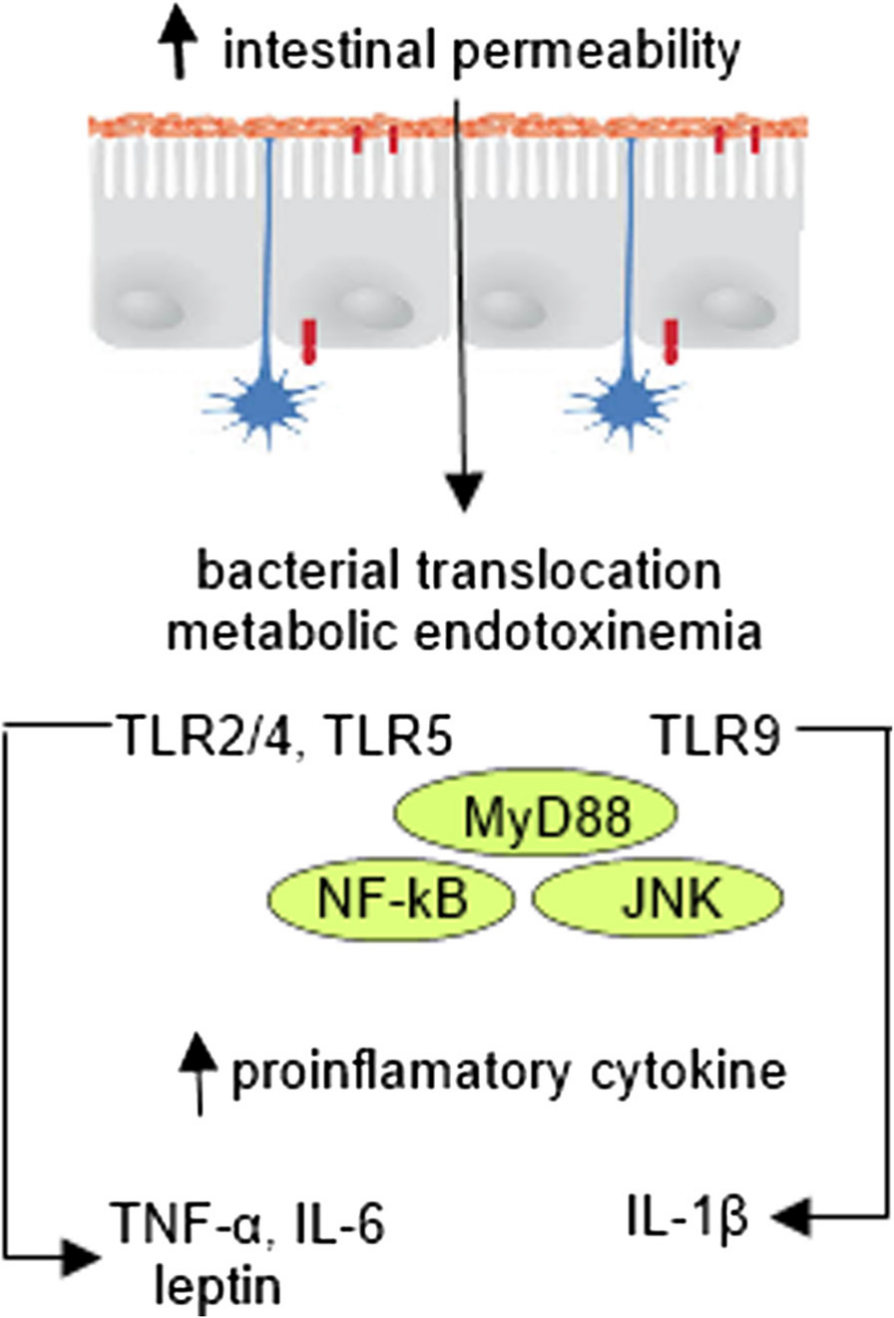
Interaction between gut microbiota, host innate immunity and metabolic inflammation

These findings directly demonstrate that modulation of the immune system is integrated with pathogen-sensing systems (e.g. TLRs) and support the emerging view that the gut microbiota contributes to the inflammation and metabolic disease (Fig. 2).

### Increased intestinal permeability

The result of the interaction of epithelial cells with symbiotic physiological microflora is formation of pre-epithelial film that consists of a layer of molecules of mucus secretory IgA, immune cells, microcolonies of obligate bacteria, enzymes and metabolites of microorganisms and the host [73]. This barrier closes the way to specific receptors on the epithelium for the living cells of harmful microflora and its toxins.

There is also growing interest to gut microbiota and intestinal mucus layer interlinks in the context of obesity and associated diseases. Several studies have confirmed this interaction, including a recent one showing that TM-IEC *C1galt*(-/-) mice with altered intestinal architecture have impaired gut microbiota composition with inverse shifts in the abundance of the phyla *Bacteroidetes* and *Firmicutes*. These knockout mice due to the impairment in mucus glycosylation have an elongated gastrointestinal tract with deeper ileal crypts, a small increase in the number of proliferative epithelial cells and thicker circular muscle layers in both the ileum and the colon [74]. Kashyap et al. [75] mentioned that modification of carbohydrate landscape of the distal gut in *Fut2(-/-)* mice that lack fucosylated host glycans can alter the fecal composition and function of resident microbes as compared to Fut2(+) control mice. Thus, the mucus layer not only affects gut architecture, but also plays a role in the regulation of gut microbiota composition and intestinal inflammation. Nevertheless key mechanisms linking intestinal mucus and gut microbiota are not fully elucidated.

Some lines of experimental evidence suggest that HFD may affect epithelial integrity due to changes in the distribution and localization of Zonula Occludens-1 (ZO-1) and Occludin (two tight junction proteins) in intestinal tissue leading to impaired gut permeability and low-grade systemic inflammation [53, 76, 77]. A recent study demonstrated that HFD mice, as compared to the control diet, have a reduced trans-epithelial resistance and mRNA expression of zona occludens 1 by 38 % (*P* < 0.001) and 40 % (*P*  =  0.025), respectively. Parallel to alteration of intestinal permeability, 6.6-fold elevation of TNF-α mRNA (*P*  =  0.037) expression in proximal colon was observed [78].

Some bacterial strains, such as *Akkermansia muciniphila*, enhance mucosal defense against pathogenic microorganisms by increasing mucin production and secretion of antimicrobial peptide regenerating islet-derived 3-γ (RegIII-γ). The amount of this substance is significantly decreased when high growth rate symbiotic bacteria effectively compete for food and adhesion sites [79].

### Cross-talk between gut microbiota and endocannabinoid (eCB) system

The endocannabinoid (eCB) system is a complex of several bioactive lipids, enzymes and different types of receptors [80]. Most-studied of the lipids are N-arachidonoylethanolamide (anandamide; AEA) and 2-arachidonoylglycerol (2-AG) [81]. Monoacylglycerol lipase (MAGL) and fatty acid amide hydrolase (FAAH) are primary enzymes that regulate production and degradation of AEA and 2-AG, respectively, from cell membrane phospholipids after cell stimulation [76]. After releasing, eCBs interact with G_i/o_-coupled receptors CB1and CB2, which are also targeted by the principal active component of *Cannabis sativa*, ∆9-tetrahydrocannabinol [82].

Several studies have confirmed that eCB plays a key role in the regulation of energy homoeostasis and in the control of lipid and glucose metabolism at several levels [83, 84]. Obesity is associated with hyperactivity of eCB as a result of dysregulation which is characterized both by increased eCB levels and CB1 activity and decreased levels of enzymes in a species- and tissue-dependent manner [85]. This dysregulation leads to the unbalanced energy intake, contributes to the excessive intra-abdominal fat accumulation and is associated with the development of metabolic alterations observed in obesity and T2D [86]. Recent studies suggested that both *CB1* (-/-) knockout mice and animals with pharmacological inhibition of CB1 by SR141716 are resistant to diet-induced obesity [82–84]. *CB1* (-/-) mice are lean due to development of hypophagia and reduced spontaneous caloric intake [87, 89]. Phenotypically mice lacking CB1 have reduced a total fat mass and decreased body weight, as compared to their WT littermates [87]. Administration of novel potential anti-obesity drug SR141716 (10 mg) induces a transient reduction of food intake (-48 % on week 1) and a marked but sustained reduction of body weight (-20 %) and adiposity (-50 %) of DIO mice [88]. This effect is negligible in *CB1*(-/-) mice, which confirms the implication of CB1 receptors in the activity of the compound [89]. Conversely, activation of CB1 receptors by intrahypothalamic injection of anandamide induces significant hyperphagia [90]. Furthermore, overexpression of CB1 or its specific activation in the liver leads to accumulation of long-chain ceramides in the liver that appear to mediate eCB-induced hepatic insulin resistance [91] and development of hyperinsulinaemia as a result of reduced insulin clearance [92].

In a recent study, Cani et al., demonstrate that gut microbiota modulate intestinal eCB system tone. Specific changes in gut microbiota in germ-free mice and in mouse models of bacterial–host interactions (HFD, treatment with anti- or probiotics) lead to significant selective decrease of CB1 mRNA expression in the colon, as compared to small intestine, and thereby regulate gut permeability and plasma LPS levels [93]. In this study, no significant modulation of CB2 mRNA expression is observed. At the same time, another group found that administration of *Lactobacillus acidophilus* increases CB2 receptor expression in the colon in mice [94]. Interestingly, specific modulation of gut microbiota with prebiotics in *ob/ob* mice reduces CB1mRNA expression in adipose tissue, decrease plasma LPS levels and increase adipocyte differentiation and lipogenesis. These data indicate that gut microbiota determine adipose tissue physiology through LPS-eCB system regulatory loops and may have critical functions in adipose tissue plasticity during obesity [93].

### Altered bile acids metabolism and BSH activity

**Fig. 3:**
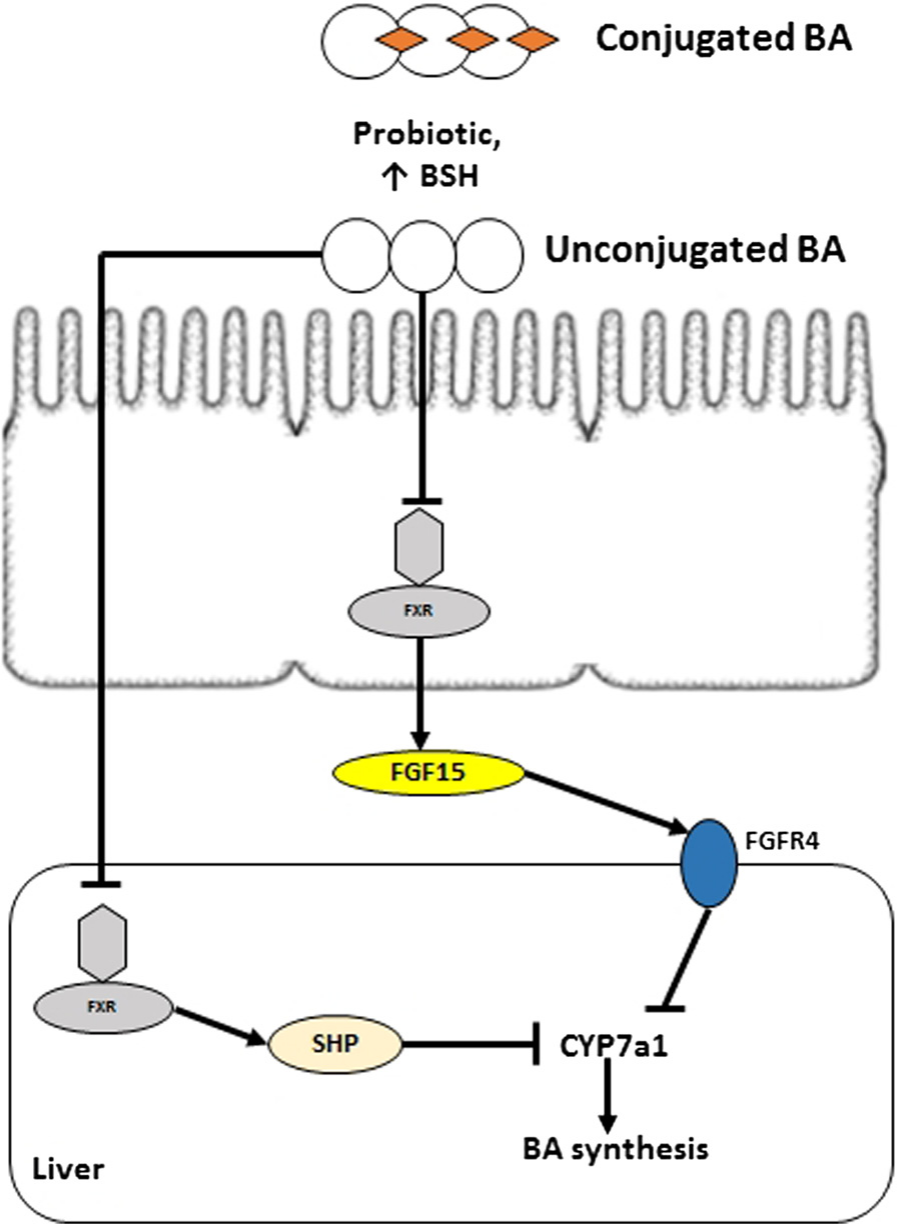
Mechanism of bile acids synthesis regulation. Impact of gut microbiota

Bile acids act as signaling molecules and activate nuclear bile acids (BA) receptor, called farnesoid X receptor (FXR), and the G-protein coupled receptor TGR5, thereby regulating energy and hepatic lipid and glucose metabolism [95, 96]. The FXR is strongly expressed in the bile acid excretion (liver) and absorption (intestine) regions. Activation of FXR induces expression of small heterodimer binding partner (SHP) and inhibits its activation of the CYP7A1 – the first and rate-limiting enzyme of BA synthesis [97]. FXR-induced FGF15 (human ortholog of FGF19) that originates in the small intestine represses hepatic bile acid synthesis through FGF receptor 4 (FGFR4) expressed in the liver, or alternatively, by activating SHP [98]. Those FXR-dependent FGF15/FGFR4 gut-liver signaling pathway that cooperate with hepatic SHP maintain the bile acid synthesis and entero-hepatic circulation (Fig. 3), but also play a key role in the control of hepatic *de novo* lipogenesis, VLDL triglyceride export and plasma triglyceride turnover [99].

A different study discussed the impact of gut microbiota modulation on BA synthesis. Administration of VSL#3 probiotics promotes ileal BA deconjugation with subsequent fecal BA excretion in mice. These events are associated with changes in ileal BA absorption and increased hepatic BA neosynthesis via downregulation of the gut-liver FXR-FGF15 axis (Fig. 3). Treatment with a FXR agonist normalized fecal BA levels in probiotic-administered mice, whereas probiotic induced alterations in BA metabolism are abolished in FXR- and FGF15-deficient animals [100].

This study shows that the principal site of protective bile acid signaling against lipid accumulation is located in the liver and not in the intestine. Using organ-specific *Fxr* knockout mice fed a 1 % cholesterol diet for 28 days, authors observed elevated triglycerides and bile acid levels with strong lipid accumulation, characterized by larger vacuoles in hepatic *Fxr -/-* sections. At the same time, intestinal studies of *Fxr -/-* mice show no histological difference and maintain normal serum cholesterol and bile acid levels, as compared to WT controls [101].

Several recent studies in mice mentioned that alteration of the gut microbiota changes host bile acid composition. It was found that in germ-free mice, a large proportion of the bile acid profile consisted of tauro-b-muricholic acid (TβMCA) (34.5 % vs 1.8 % of the plasma profile in conventionally raised) [102]. Bacterial suppression through antibiotic treatment induced a similar shift with taurine-conjugated bile acids with increases in tissue bile acid profiles. Notably, antibiotic treatment can antagonize the intestinal FXR/FGF15 as well [103, 104].

In a recent animal study, reduction of BSH activity by antibiotic or tempol treatment of HFD-fed mice was shown to prevent NAFLD by modulating gut microbiota and altering metabolism of bile acids, with a notable increase of the FXR antagonist T-β-MCA, which inhibited FXR signaling in the intestine. Compared with control mice, animals with intestine-specific *Fxr* disruption had reduced hepatic triglyceride accumulation by 50 % in response to a HFD. Inhibition of intestinal FXR signaling elicits an improvement in mitochondrial function and results in decreased serum ceramide levels which downregulate hepatic SREBP1c and CIDEA expression. This in turn, results in decreased hepatic steatosis. Interestingly, administration of C16:0 ceramide in antibiotic-treated mice fed a HFD reversed hepatic steatosis [105].

Joyce et al., using a controlled expression system, showed that bacterial BSH mediates a microbe-host dialogue that regulates lipid metabolism and weight gain in the host. Colonization of the gastrointestinal tract by *E. coli* MG1655 as a delivery vector capable of expressing cloned BSH genes leads to significantly altered plasma bile acid composition and regulates transcription of key genes involved in lipid metabolism (Ppar-γ, Angptl4) and gastrointestinal homeostasis (RegIIIγ) in mice. High-level expression of BSH in conventionally raised mice results in a significant reduction in host weight gain, plasma cholesterol, and liver triglycerides [106]. Because numerous well-known probiotics exhibit BSH activity [107], this may partially account for their metabolic effects.

TGR5 (also known as GPBAR1, M-BAR and BG37) is a G-protein coupled receptor expressed in brown adipose tissue and muscle where its activation by secondary lithocholic bile acids with subsequent induction of the enzyme 2-iodothyronine deiodinase triggers an increase in energy expenditure. This enzyme converts inactive thyroxine (T4) to tri-iodothyronine (T3), resulting in an increase in metabolic rate and energy expenditure. Stimulation of TGR5 attenuates diet-induced obesity [96]. Thomas et al. demonstrated that TGR5 is expressed in L-cells and its activation induces intestinal GLP-1 release, leading to the improved liver and endocrine pancreatic function and enhanced glucose tolerance in HFD mice. These data show that the TGR5 signaling pathway is critical in regulating intestinal GLP-1 secretion in vivo and suggest that pharmacological targeting of TGR5 may constitute a promising treatment of metabolic disorders [108].

In conclusion, bile acids have a bacteriostatic activity and diet enriched with fats changes the bile acid composition, which influences the conditions for gut microbial environment and causes dysbiosis [109]. By modifying bile acid metabolism and FXR/TGR5 signaling, gut flora could therefore contribute indirectly to the pathogenesis of metabolic syndrome, and manipulation of its composition can be a promising novel drug target for the treatment of the obesity-associated diseases.

## Conclusion

The review describes the underlying mechanisms of the association of microbiota with the metabolic processes and obesity of the host organism. The altered microbiota may be an environmental factor of the obesity development. Among links between dysbiosis and obesity are downregulated activity of FIAF and AMPK, decreased consumption of vitamins and biologically active compounds, impaired production of SCFAs, increased inflammation, gut permeability and endotoxemia, altered LPS-eCB system regulatory loops and bile acids metabolism. Probiotic therapy is proposed as a promising strategy in the management of metabolic disorders and obesity because of its restoration of microflora composition and maintenance of human health via diverse aforementioned mechanisms.

## Abbreviations

2-AG: 2-arachidonoylglycerol
ACC: acetyl-CoA carboxylase
AEA: N-arachidonoylethanolamide
AMPK: AMP-activated protein kinase
BA: bile acids
BSH: bile salt hydrolase
ChREBP: carbohydrate responsive element binding protein
CTP1: carnitine-palmitoyltransferase 1
DC: dendritic cells
eCB: endocannabinoid system
FAAH: fatty acid amide hydrolase
FGF: fibroblast growth factor
FGFR4: FGF receptor 4
FIAF: fasting-induced adipose factor
FXR: farnezoid X receptor
GLP-1: glucagon-like peptide-1
GPR: G protein-coupled receptors
HFD: high fat diet
IRAK: interleukin 1 receptor associated kinases
Lpl: lipoprotein lipase
LPS: lipopolysaccharide
LTK: lipoteichoic acids
MAGL: monoacylglycerol lipase
MAP2K: mitogen-activated protein kinase
MyD88: myeloid differentiation primary response gene
NAFLD: non-alcoholic fatty liver disease
NIK: NF-κB-inducing kinase
PAMP: pathogen-associated microbial patterns
SCFAs: short-chain fatty acids
SHP: small heterodimer binding partner
SREBP-1: sterol responsive element binding protein
T2D: type 2 diabetes
TAB1: TAK-binding protein 1
TAK-1: TGF-β-activated kinase 1
TGF-β: transforming growth factor β
TLR-4: toll-like receptor-4
TRAF6: TNF receptor associated factor 6
TβMCA: tauro-b-muricholic acid
VAT: visceral adipose tissue
ZO-1: Zonula Occludens-1
